# Dermatomyositis as the First Manifestation of Nasopharyngeal Carcinoma—A Rare Case Report

**DOI:** 10.3390/medicina61020334

**Published:** 2025-02-14

**Authors:** Bojana Knežević, Tatjana Radević, Milan Petronijević

**Affiliations:** Military Medical Academy, Faculty of Medicine, University of Defense, Crnotravska 17, 11000 Belgrade, Serbia; tradevic@hotmail.com (T.R.); milanpetronijevic@yahoo.com (M.P.)

**Keywords:** dermatomyositis, nasopharyngeal carcinoma, case report, treatment, autoimmune diseases, paraneoplastic syndrome, Epstein–Barr virus, malignancy screening

## Abstract

Nasopharyngeal carcinoma (NPC) with paraneoplastic dermatomyositis (DM) is an exceptionally rare clinical phenomenon, particularly among European populations. This case report details a 46-year-old woman initially diagnosed with DM, later confirmed to have NPC. Such an association is more frequently documented in Asian populations, highlighting its unique presentation in this case. The patient first developed symptoms in December 2016, which progressed significantly by spring 2017, manifesting as progressive proximal muscle weakness, characteristic skin changes, and elevated muscle enzyme levels. Diagnostic workup, including electromyography and biopsy, confirmed DM. Persistent symptoms and secondary DM suspicion prompted further malignancy screening, which identified undifferentiated NPC with strong Epstein–Barr virus RNA positivity. Multimodal treatment comprising corticosteroids, hydroxychloroquine, chemotherapy, and radiotherapy led to temporary symptomatic improvement. Despite initial success, the patient’s condition deteriorated, and she passed away by the end of 2018. This case underscores the importance of comprehensive malignancy screening in DM patients, considering rarer cancers like NPC even in non-endemic regions. It emphasizes the role of multidisciplinary care and adherence to international guidelines for managing such complex cases. Recognizing NPC-associated DM remains critical for early intervention and tailored therapeutic approaches to improve clinical outcomes and survival.

## 1. Introduction

Nasopharyngeal carcinoma (NPC) is a rare malignant squamous-cell carcinoma originating from the epithelial lining of the nasopharynx [1,2,3,4]. The disease shows distinct patterns of regional, racial, and gender prevalence. According to 2022 data, global age-standardized incidence rates (ASIRs) vary widely, with rates ranging from 2.2 per 100,000 in Asia to 0.6 per 100,000 in Europe and 0.57 in Serbia [5]. Based on data from the International Agency for Research on Cancer, NPC represented only 0.7% of all diagnosed tumors globally in 2018 [5,6]. The disease is particularly prevalent in East and Southeast Asia, especially in southern China [7]. Incidence rates are notably higher in males than in females, with a male-to-female ratio of approximately 2.5 in China in 2015 [8] and a ratio of 1.65 in Serbia in 2022 [5].

Dermatomyositis (DM) is a rare autoimmune connective tissue disease, primarily characterized by skin manifestations [4,9,10]. It involves noninfectious inflammatory damage to the skin and predominantly affects the proximal muscles, especially those in the shoulder and pelvic girdles [11]. Dermatomyositis can be associated with an underlying malignancy in approximately 18–32% of cases, often serving as a paraneoplastic marker for cancers [12].

This paper aims to present the case of a female patient diagnosed with secondary dermatomyositis associated with NPC, both of which are considered rare, particularly in females. In Serbia, both NPC and DM are exceptionally rare, making this case particularly significant due to the extreme rarity and the unique interplay between these two conditions.

## 2. Case Presentation

In December 2016, a 46-year-old Caucasian female patient began experiencing initial symptoms that included pain and a sensation of blockage in her right ear. By February 2017, her condition progressed, and she developed polyarthritis affecting the small joints of her hands. This joint pain was accompanied by notable skin changes on the extensor surfaces of her hands, which later extended to her thighs and shoulders.

In March 2017, the patient began experiencing significant muscle weakness, primarily affecting the proximal muscles of both her shoulder and pelvic girdles. This was accompanied by swelling in the neck lymph nodes and difficulty swallowing solid foods, which prompted her to seek medical attention. She was admitted to a local hospital in Serbia, where she remained from 4th to 22nd April 2017. During this hospitalization, the medical team conducted a thorough virological evaluation to identify potential infectious causes of her symptoms. Tests were performed for Epstein–Barr virus (EBV), toxoplasma, cytomegalovirus (CMV), Lyme disease, hepatitis B and C (HBV, HCV), and HIV. All results came back negative, effectively ruling out an infectious etiology for her condition (Table 1). Despite the absence of an identifiable infectious cause, the doctors at the local hospital were initially uncertain about the underlying issue and opted to treat her empirically with antibiotics. This decision was based on the presumption of possible otitis media and sinusitis, which are common conditions that can sometimes present with nonspecific symptoms. However, this treatment approach did not lead to any improvement in her condition.

Further diagnostic investigations were conducted, including a chest X-ray and abdominal ultrasound, both of which yielded normal results. However, laboratory tests revealed persistently elevated inflammatory markers and a significantly high creatine kinase (CK) level of 1047 (Table 1), which is indicative of muscle damage. Given these findings, an electromyography (EMG) was performed on both her upper and lower extremities. The EMG results showed myopathic potentials and spontaneous fibrillations, a pattern consistent with polymyositis, a rare inflammatory muscle disease. Based on these findings, the patient was referred to a rheumatologist for specialized management and further treatment.

**Figure 1:**
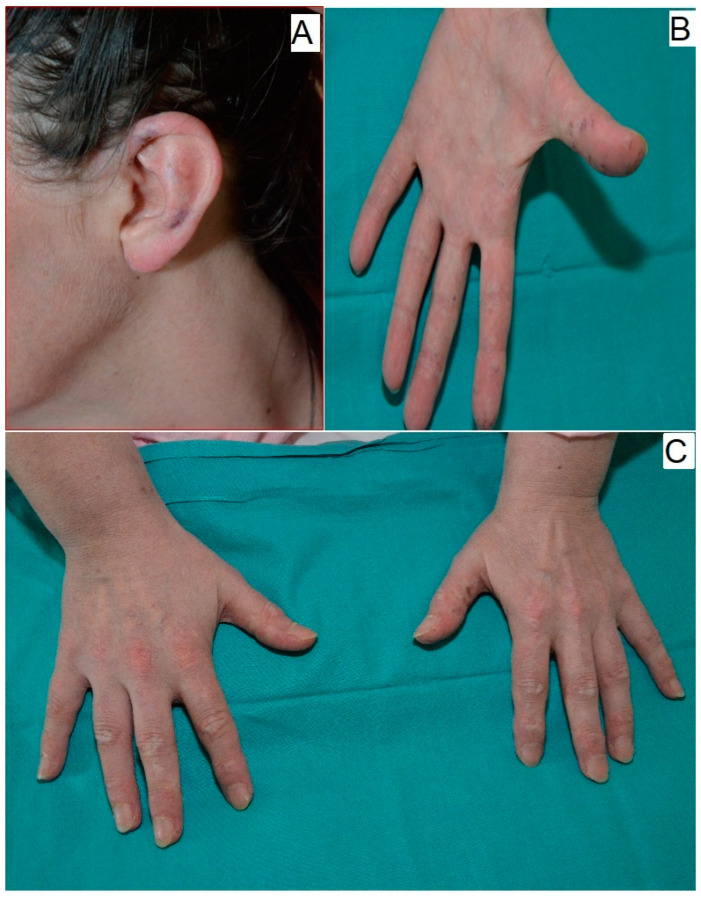
Skin findings on admission to MMA. Physical examination revealed skin manifestation of dermatomyositis. (**A**) Ear, (**B**) thumbs, and (**C**) Gottron papules on the hand.

In April 2017, the patient was admitted to the Rheumatology Clinic at the Military Medical Academy (MMA) in Belgrade, Serbia, for further evaluation and management. The patient presented with dermatologic and musculoskeletal features consistent with a systemic inflammatory myopathy, including erythematous changes involving the trunk, ears (Figure 1A), and extremities (Figure 1B), accompanied by prominent telangiectasia. Additional findings included a heliotrope rash on the upper eyelids and Gottron’s papules on the dorsal hands (Figure 1C), alongside proximal muscle weakness affecting the shoulder and pelvic girdles, which severely limited head control. Bilateral lymphadenopathy posterior to the sternocleidomastoid muscles, characterized by firm, enlarged nodes, raised suspicion for an underlying systemic inflammatory or autoimmune process. Initial diagnostic steps included a cervical lymph node ultrasound and a fine-needle aspiration biopsy (FNAB) on 3 May, which revealed cytologic evidence of malignancy, prompting recommendations for excisional biopsy with histopathological analysis.

Subsequent evaluations, spanning from 4 to 26 May 2017, were guided by concerns for paraneoplastic dermatomyositis. Multislice computed tomography (MSCT) of the chest, abdomen, and pelvis (4 May) was followed by gynecologic examination (9 May) to exclude pelvic malignancy. Further investigations included esophagogastroduodenoscopy (EGDS), abdominal/pelvic ultrasound, and repeat cervical lymph node biopsy (11 May); endoscopic ultrasonography (15 May) to assess gastrointestinal involvement; and breast ultrasound with concurrent MSCT of the head/neck (18 May), which identified a nasopharyngeal lesion later biopsied (19 May). An echocardiogram (26 May) evaluated cardiac involvement. This systematic, chronologically staged workup integrated clinical, imaging, and histopathologic data, ultimately linking the dermatomyositis phenotype and lymphadenopathy to an underlying malignancy.

Laboratory investigations were consistent with a diagnosis of inflammatory myopathy and provided further insight into the systemic inflammatory nature of her condition. Biohumoral markers of inflammation were elevated, including an erythrocyte sedimentation rate (ESR) of 43 mm/h and a C-reactive protein (CRP) level of 14 mg/L. Hematological analysis revealed normocytic anemia, accompanied by leukocytosis. Liver enzyme levels were notably elevated, with aspartate transaminase (AST) at 319 U/L and alanine transaminase (ALT) at 122 U/L. Muscle enzyme tests showed marked elevations, with CK reaching 5632 U/L and myoglobin at 308 ng/mL, indicating active muscle breakdown and inflammation. Lactate dehydrogenase (LDH) was also elevated at 501 U/L (Table 1).

Immunological assays revealed a positive antinuclear antibody (ANA) test with a speckled pattern at a 3+ intensity, which is often associated with systemic autoimmune diseases (Table 1). Tests for rheumatoid factor (RF), double-stranded DNA (dsDNA) antibodies, and extractable nuclear antigen (ENA) panel were negative (Table 1), helping to exclude other rheumatologic conditions such as systemic lupus erythematosus and mixed connective tissue disease. Anti-transcriptional intermediary factor 1 gamma (TIF-1γ) autoantibodies were not tested in this case, as the assay was unavailable at our institution (MMA) during the patient’s evaluation and remains inaccessible to date.

The patient met the diagnostic criteria for dermatomyositis and began treatment with three pulse doses of intravenous methylprednisolone at 500 mg, followed by daily oral methylprednisolone at a dose of 1 mg/kg. Following the initiation of this regimen, the patient showed clinical improvement, including the resolution of skin lesions and normalization of inflammatory markers. However, significant muscle weakness persisted, raising suspicion of secondary dermatomyositis and prompting further investigation to assess for an underlying malignancy, a known potential trigger for paraneoplastic dermatomyositis.

A comprehensive diagnostic workup was conducted, including echocardiography, abdominal and pelvic ultrasound, multislice computed tomography (MSCT) of the chest and abdomen, esophagogastroduodenoscopy (EGDS), and gynecological examination, all of which were negative for solid tumors. However, ultrasound of the neck revealed multiple pathologically enlarged lymph nodes bilaterally along the major cervical vessels, suggestive of a possible lymphoproliferative or metastatic process.

A biopsy of one of the enlarged cervical lymph nodes was performed, revealing undifferentiated nasopharyngeal carcinoma. In situ hybridization demonstrated high levels of Epstein–Barr virus (EBV) RNA (EBER) within the tumor cells, establishing a link between the malignancy and EBV infection, which is commonly associated with nasopharyngeal carcinoma.

**Figure 2:**
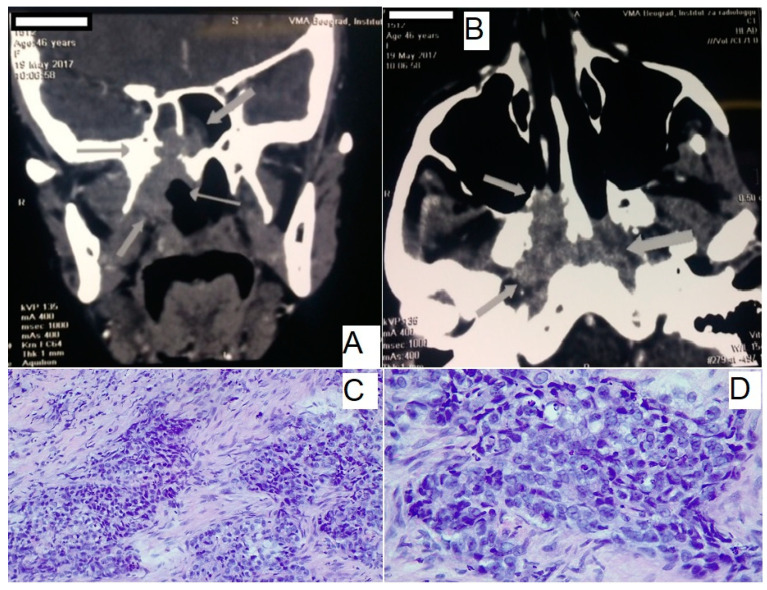
Clinical and diagnostic imaging findings. (**A**,**B**) MSCT of head and neck, showing axial and coronal views of the tumor mass. Arrows are showing tumor mass and margins of tumor. (**C**,**D**) Pathohistological analysis of the tumor biopsy, confirming the diagnosis of undifferentiated nasopharyngeal carcinoma. Hematoxylin and eosin staining reveals characteristic features, including large pleomorphic nuclei, prominent nucleoli, and a syncytial growth pattern. MSCT, Multislice computed tomography.

Additionally, an MSCT scan of the head and neck was performed (Figure 2A,B), which provided a detailed assessment of the tumor’s extent. The imaging revealed a mass in the epipharyngeal and nasopharyngeal regions, extending into the posterior choanae, with invasion into the sphenoid sinus and erosion of the intersphenoid septum. Posteriorly, the tumor infiltrated the prevertebral musculature without compromising the C1 vertebra.

Further evaluation by an otolaryngology specialist through nasopharyngoscopy identified a substantial tumor mass located on the upper wall of the epipharynx. A biopsy of this lesion confirmed the diagnosis of undifferentiated NPC (Figure 2C,D).

This constellation of findings established a diagnosis of EBV-associated undifferentiated NPC, likely as the underlying malignancy associated with dermatomyositis. This discovery has important implications for both the patient’s oncologic and rheumatologic management, warranting a multidisciplinary approach to treatment involving oncology, rheumatology, and otolaryngology teams.

Considering that patients with DM have a 5–7 times higher risk of malignancies—and that DM can present before, simultaneously with, or after a cancer diagnosis—screening for malignancies was and still is mandatory in patients with DM. The patient’s case was reviewed by a multidisciplinary tumor board for head and neck malignancies. Staged as T3N2M0—indicating a locally advanced tumor with significant lymph node involvement, but no distant metastasis—the board recommended combined radiotherapy and chemotherapy. Treatment began in early June 2017, incorporating radiotherapy (66Gy) targeting the primary tumor and lymph nodes and five cycles of chemotherapy following the PF protocol (cisplatin and 5-fluorouracil (5-FU)). Concurrently, rheumatologic management included prednisone (40 mg daily, tapered) and hydroxychloroquine (400 mg/day) to manage dermatomyositis symptoms. This integrated approach aimed to achieve both tumor control and symptom management associated with her paraneoplastic syndrome.

At her most recent follow-up in April 2018, with both the otolaryngology and rheumatology departments at the MMA, the patient reported a subjective sense of well-being. Physical examination revealed only mild residual weakness in the neck musculature, while all dermatologic manifestations had completely resolved, indicating a positive response to both oncologic and rheumatologic treatments.

However, by the end of 2018, the patient’s condition reportedly deteriorated, as noted by her husband. She ultimately passed away in a regional healthcare facility in Prokuplje, Serbia. Her case highlights the complexity of managing paraneoplastic dermatomyositis associated with advanced nasopharyngeal carcinoma, underscoring the need for a coordinated, multidisciplinary approach to address both oncologic and autoimmune manifestations effectively.

## 3. Discussion

Nasopharyngeal carcinoma is classified by the World Health Organization into three histological types: keratinizing squamous cell carcinoma, non-keratinizing carcinoma (differentiated and undifferentiated), and basaloid carcinoma [13]. In high-incidence regions, undifferentiated carcinoma dominates, comprising over 95% of cases and offering relatively favorable survival outcomes [3,7]. NPC’s etiology is multifactorial, involving genetic predisposition, ethnicity, diet, environmental factors, tobacco use, and EBV infection [2,14,15]. These factors highlight its origin as a complex interplay of EBV infection, genetic vulnerability, and environmental exposures.

Dermatomyositis affects both children and adults, with a prevalence of ~1/100,000 and higher incidence in women [9,10]. Diagnosis hinges on characteristic skin rashes (e.g., “V-shaped” erythema with photosensitivity/pruritus), muscle weakness, elevated enzymes, electromyography abnormalities, and biopsy findings [16]. Symptoms range from fatigue to severe dyspnea/dysphagia; unintended weight loss may signal malignancy [17].

The pathogenesis of dermatomyositis remains largely unclear, with several theories proposed, especially regarding its association with malignancy. The hormonal theory suggests that tumors secrete biologically active polypeptides that disrupt homeostasis, leading to endocrine-like syndromes observed in DM and other paraneoplastic conditions [18]. In contrast, the immunological theory suggests that DM results from an immune response to tumor antigens, with antibodies cross-reacting with similar structures in healthy tissues, thereby triggering an autoimmune attack on muscle and skin. This cross-reactivity may drive the inflammatory processes characteristic of DM, linking both autoimmune and oncologic factors in the disease’s pathogenesis [18].

The diagnosis of dermatomyositis adheres to Bohan and Peter criteria (1975) [16], requiring proximal symmetrical weakness and hallmark skin findings (heliotrope rash, Gottron’s papules, extensor surface dermatitis). Muscle biopsy findings, such as perifascicular atrophy and inflammation, further confirm the diagnosis [16]. For our patient, the updated EULAR/ACR Classification Criteria [19] were also applied.

Our approach adhered to ESMO-EURACAN Clinical Practice Guidelines for NPC [20], supplemented by the Chinese Society of Clinical Oncology (CSCO) recommendations for NPC diagnosis and management [21]. These internationally recognized guidelines offered a robust framework for addressing this rare European case of NPC-associated DM.

By combining the CSCO [21] and ESMO-EURACAN [20] guidelines, we ensured a comprehensive diagnostic approach incorporating advanced imaging techniques like MSCT for tumor assessment, biopsy for pathological confirmation, and EBV-specific testing using in situ hybridization.

This case highlights the importance of adapting global protocols to address rare and complex presentations, such as NPC-associated DM in a European patient. Leveraging guidelines from regions with high NPC prevalence allowed us to navigate the unique challenges of this ultra rare diagnosis effectively.

Elevated serum muscle enzymes, such as aldolase, and LDH, are markers of muscle necrosis, rising in 70–90% of DM cases. However, normal levels do not rule out the diagnosis, as enzyme activity may vary. Additional diagnostics, including EMG and imaging, help distinguish DM from other inflammatory and myopathic conditions, offering a more complete clinical picture [11].

Malignancy is identified in approximately 18–32% of DM cases, with common associations including ovarian, bronchial, breast, and head and neck cancers, as well as lymphomas [12]. DM symptoms may precede, follow, or coincide with cancer detection [12], sometimes emerging up to five years before malignancy diagnosis [22]. The incidence of DM associated with NPC is rare, occurring in about 1 in 1000 cases [23].

The link between NPC and paraneoplastic syndromes like DM is well-documented, though the underlying mechanisms remain unclear [24]. The first case was reported in 1969 [25], this association has been supported by numerous studies. For instance, Chan found 41% of DM cases in Singapore were associated with NPC, indicating a notable link in this population [26]. Similarly, a Chinese study reported malignancy in 20.3% of DM patients, with NPC comprising 78.5% of these cases, highlighting a strong connection in East Asian populations. This relationship may be influenced by genetic, environmental, and viral factors, particularly EBV, which plays a role in NPC pathogenesis [27].

Antibodies, particularly anti-TIF-1γ, have gained substantial importance in the diagnostic and prognostic evaluation of paraneoplastic DM. Anti-TIF-1γ is the most common malignancy-associated myositis-specific antibody (MSA), with a reported prevalence of cancer ranging from 38% to 80% in patients with this autoantibody [28,29]. Breast cancer is the most frequently associated malignancy, followed by ovarian, colorectal, and lung cancers, as well as lymphomas [30,31]. The diagnostic specificity and sensitivity of anti-TIF-1γ vary depending on the type of malignancy [31], which underscores its clinical relevance in guiding appropriate therapeutic strategies. Unfortunately, at the Military Medical Academy (MMA), testing for anti-TIF-1γ autoantibodies was not available at the time of diagnosis and remains unavailable currently. This highlights a critical gap in diagnostic capabilities, given the antibody’s established importance in identifying and stratifying cancer risk in DM. Compared to other autoantibodies, such as anti-Jo-1 or anti-Mi-2, anti-TIF-1γ exhibits unique patterns of association with malignancy. For instance, while anti-Jo-1 is often linked to interstitial lung disease [32], anti-TIF-1γ is strongly associated with a higher incidence of cancer in DM patients [33]. These differences in autoantibody profiles provide critical insights for prognostic evaluation and personalized treatment approaches, enabling clinicians to better assess the risk of malignancy in DM patients. Recent studies have further elucidated the mechanisms underlying the association between anti-TIF-1γ autoantibodies and cancer-associated DM [31,33,34]. It has been hypothesized that TIF1-γ functions as a tumor autoantigen, with tumors in paraneoplastic anti-TIF1-γ-positive patients showing increased genetic alterations, such as mutations and loss of heterozygosity in TIF1 genes [33]. The high expression of TIF1-γ in tumor, muscle, and skin tissues may explain the immune cross-reactivity that leads to the clinical manifestations of DM. Specifically, aberrant TIF1-γ in cancer cells may generate neoantigens targeted by the immune system, which cross-react with wild-type TIF1-γ in muscle and skin. This immune response can either eradicate tumor cells without clinical symptoms or, if unsuccessful, result in tumor progression and the development of DM [33]. The high concentration of TIF1-γ antigens in muscle and skin tissues likely contributes to the clinical presentation of DM in these patients. These findings highlight the dual role of anti-TIF1-γ autoantibodies as both biomarkers of malignancy and key players in the pathogenesis of paraneoplastic DM [33], offering valuable insights into tumor biology and immune-mediated disease mechanisms.

In addition to the aforementioned biomarkers, novel parameters such as the neutrophil-to-lymphocyte ratio (NLR) and platelet-to-lymphocyte ratio (PLR) have emerged as valuable predictors of survival and quality of life in patients. A study of 427 primary NPC patients treated with radiotherapy found that elevated pre-treatment biomarkers—NLR and PLR were independently associated with poorer overall and progression-free survival, emphasizing the prognostic value of these accessible biomarkers as a complement to cancer staging and for guiding more personalized treatment strategies [35]. Similarly, Cocuzza et al. (2018) found that NLR and PLR can predict dysphagia severity in NPC patients, thereby supporting the development of tailored therapeutic approaches to improve patient quality of life [36].

EBV plays a pivotal role in the pathogenesis of NPC [37], where its genome and viral proteins are consistently detected in tumor cells, contributing to oncogenesis by altering cellular proliferation and evading apoptosis [38]. Chen et al. also showed a positive association of EBV with DM and NPC [38]. Moreover, EBV has been implicated in triggering autoimmune responses that may lead to DM, possibly via mechanisms such as molecular mimicry and immune dysregulation, which promote the production of autoantibodies and pro-inflammatory cytokines [39]. In patients with DM, particularly those with associated NPC, elevated levels of EBV-specific antibodies have been observed, underscoring the virus’s dual role in driving malignancy and potentially initiating autoimmune processes [37,40]. However, Chen’s extensive study involving 172 patients found no significant difference in overall survival between patients with DM and higher EBV titers than those without DM and with lower EBV titers [40]. This complex interplay between EBV-induced oncogenesis and immune activation highlights the importance of considering EBV not only as a biomarker for NPC but also as a potential contributor to the autoimmune phenomena seen in DM.

A systematic literature review and network meta-analysis of 95 studies involving 16,010 DM patients identified 303 NPC cases, yielding a 3.3% prevalence of NPC among DM patients, with national estimates ranging from 0.1% to 36.5% [41]. Nearly half of the studies were conducted in Asia [41], where NPC is more prevalent, especially in Southeast Asia and Southern China [42]. NPC-associated DM is rare in Western populations [43], where lung, breast, ovarian, and colorectal cancers are more commonly linked to DM [12,44,45,46,47]. European cases remain extremely rare, as evidenced by a clinical case from Italy [46] and another from the UK documenting the first known NPC-associated DM cases in the UK [47].

This regional disparity highlights environmental, genetic, and viral influences, such as the high prevalence of EBV in NPC-endemic areas. Tailored screening strategies and heightened clinical suspicion are crucial for diagnosing NPC in DM patients, even in non-endemic areas. Our patient exemplifies the importance of thorough diagnostic evaluations, which led to the confirmation of NPC alongside secondary DM in a European context.

This connection between NPC and DM suggests that NPC may increase susceptibility to DM, while DM can serve as a predictive marker for NPC in specific cases. Patients with NPC-associated DM typically exhibit skin changes followed by proximal muscle weakness [48]. Malignancy-associated myopathies, including those linked to NPC, are typically more treatment-resistant and carry a poorer prognosis than non-malignancy-related myopathies [16,49]. This resistance is largely due to tumor-related immune dysregulation, in which the underlying malignancy induces a persistent autoimmune response characterized by chronic inflammation, muscle damage, autoantibody production, and the activation of autoreactive T cells that are not adequately controlled by conventional treatments [48,50,51]. Consequently, these findings underscore the importance of routine cancer screening for DM patients—especially in high-risk populations—to facilitate a comprehensive management approach that addresses both the autoimmune component and the associated malignancy [27,52,53,54].

The effective management of cancer-associated DM involves addressing both the DM and the underlying malignancy. Standard treatment for DM begins with high-dose corticosteroids, aiming to improve muscle strength and manage extramuscular symptoms [55]. Corticosteroids are usually started at 1 mg/kg/day, maintained for 4–8 weeks until symptoms and muscle enzyme levels improve, followed by a gradual taper over 6–9 months to minimize relapse risk [56]. Severe cases may require intravenous methylprednisolone boluses before transitioning to oral corticosteroids [57].

Identifying DM in a patient should trigger further investigations for cancer, as early detection can significantly enhance treatment success and prognosis [58]. Consequently, clinicians must conduct a thorough assessment of newly diagnosed DM patients, including a detailed review of prior signs and symptoms, to evaluate the possibility of an associated malignancy [59,60]. Moreover, comprehensive cancer screening is advised within the first 3 to 5 years following disease onset—although the ideal screening strategy for newly diagnosed DM remains undefined—with improvements assessed by integrating physical examination findings, restored muscle strength, decreased fatigue, and improved skin condition. Lifelong monitoring through regular physical examinations is also recommended, particularly during the first 3 years post-diagnosis [61].

Building on the importance of early detection, the patient’s management adhered to established protocols that included initiating corticosteroid therapy—with gradual tapering based on clinical response—and targeted treatment for NPC. Management was guided by evidence-based, regionally adapted strategies per the ESMO-EURACAN Clinical Practice Guidelines [20] and CSCO Guidelines [21]. However, the advanced tumor stage at diagnosis and the prolonged diagnostic delay in identifying the malignancy likely contributed critically to the fatal outcome. These prognostic factors highlight the necessity of early detection and prompt intervention in paraneoplastic dermatomyositis, especially when linked to aggressive cancers, to mitigate poor clinical trajectories.

## 4. Conclusions

NPC with paraneoplastic DM is an exceptionally rare but distinct clinical entity. This case report highlights a 46-year-old woman in Serbia who was initially diagnosed with DM, followed by a subsequent diagnosis of NPC. Such an association is more commonly observed in Asian populations, making this case particularly noteworthy. The patient initially responded well to the treatment of both DM and NPC; however, her condition deteriorated by the end of 2018, and she passed away at a regional hospital. This case underscores the importance of vigilant diagnostic and therapeutic strategies in managing such rare associations.

## Figures and Tables

**Table 1:** Laboratory findings.

Parameters	Data from Prokuplje Hospital From 4 to 22 April 2017	Data from MMA From 26 April to 29 May 2017 at Admission	Data from MMA From 26 April to 29 May 2017 at Discharge	Data from MMA Control Check-Up 25 August 2017	Reference Range
ESR (mm/h)	12	43	12	9	0–20 mm/h
CK (U/L)	1047	5632	151	44	25–192 U/L
CKMB	N/A	113	18	12	0/25 U/L
LDH (U/L)	1357	501	232	N/A	110–240 U/L
CRP (mg/L)	N/A	14	12	0.7	<5 mg/L
Hemoglobin (g/L)	N/A	115	115	109	110–150 g/L
Leukocytes (10^9^/L)	N/A	16.4	8.74	9.7	4–10 × 10^9^/L
ALT (U/L)	N/A	122	52	42	0–45 U/L
AST (U/L)	N/A	319	39	22	0–40 U/L
Myoglobin	N/A	308	N/A	N/A	0–70 ng/mL
Tumor markers					
CEA	N/A	Within range	N/A	N/A	0–2.9 ng/mL
AFP	N/A	Within range	N/A	N/A	0–10 ng/mL
NSE	N/A	Within range	N/A	N/A	≤15 ng/mL
CYFRA21.1	N/A	Within range	N/A	N/A	≤3.5 ng/mL
CA19.9	N/A	Within range	N/A	N/A	0–37 U/mL
CA125	N/A	Within range	N/A	N/A	0–35 U/mL
CA72.4	N/A	Within range	N/A	N/A	<6.9 U/mL
CA15.3	N/A	Within range	N/A	N/A	<31.3 U/mL
ANA test	N/A	3+ (speckled pattern)	N/A	2+ (speckled pattern)	N/A
RF (IU/mL)	N/A	12	N/A	7	0–14 IU/mL
Anti-dsDNA	N/A	Negative	N/A	N/A	Negative
ENA screening	N/A	Negative	N/A	Negative	Negative
EBV	Negative	N/A	N/A	N/A	Negative
Toxoplasma	Negative	N/A	N/A	N/A	Negative
CMV	Negative	N/A	N/A	N/A	Negative
Lyme	Negative	N/A	N/A	N/A	Negative
HBV	Negative	N/A	N/A	N/A	Negative
HCV	Negative	N/A	N/A	N/A	Negative
HIV	Negative	N/A	N/A	N/A	Negative

Abbreviations: MMA, Military Medical Academy. AFP, alpha fetoprotein; ALT, aspartate aminotransferase; AST, alanine transaminase; ANA, antinuclear antibody test; Anti-dsDNA, anti-double-stranded (ds) DNA antibody; CA15.3, cancer antigen 15-3; CA19.9, cancer antigen 19-9; CA72.4, cancer antigen 72-4; CA125, cancer antigen 125; CEA, carcinoembryonic antigen; CK, creatine phosphokinase; CKMB, creatine kinase MB; CMV, cytomegalovirus; CRP, C-reactive protein; CYFRA 21-1, cytokeratin 19 fragment antigen; EBV, Epstein–Barr virus; ENA, extractable nuclear antigen; ESR, erythrocyte sedimentation rate; HBV, hepatitis B virus; HCV, hepatitis C virus; HIV, human immunodeficiency virus; LDH, lactic dehydrogenase; N/A, not applicable; NSE, neuron-specific enolase; RF, rheumatoid factor.

## Data Availability

The original contributions presented in this study are included in the article. Further inquiries can be directed to the corresponding author.
